# FORGING AND SUPPORTING INTERGENERATIONAL CONNECTIONS: THE BREADTH OF INTERGENERATIONAL SCHOLARSHIP

**DOI:** 10.1093/geroni/igac059.040

**Published:** 2022-12-20

**Authors:** Cal Halvorsen, Ling Xu, Amy Eisenstein

## Abstract

Research on intergenerational connections is wide ranging, highlighting the various ways that people from across the life course interact for mutual and community benefit. Intergenerational connections occur through both formal (e.g., community service programs) and informal (e.g., family interactions) means. Studies have shown that intergenerational connections can lead to an assortment of physical, social, psychological, and interpersonal health and well-being outcomes for younger and older people alike. This symposium will highlight the breadth of research on intergenerational connections, bringing together scholars who have used primary and secondary data to better measure, understand, and support them. The first presentation will report the results of a randomized controlled trial in Tarrant County, Texas that trained college students to connect with older adults experiencing cognitive impairment through reminiscence and digital storytelling. The second presentation will describe how intergenerational transfers of emotional and instrumental support relate to self-rated health among older Chinese adults living in Honolulu while considering the roles of gender and resilience. The third presentation will address an important question within intergenerational scholarship—how to measure intergenerational connections—by reporting the results of the newly developed and validated Intergenerational Contact survey among an online sample of younger and older adults. The fourth presentation will describe the pilot-year evaluation of the Gen2Gen Innovation Fellowship, a cohort model that supports and connects leaders of intergenerational initiatives throughout the U.S. To conclude, our discussant will place these diverse studies into context and note places for continued innovation in intergenerational scholarship to better inform the field.

